# High total serum IgE level at diagnosis was associated with a progressive decline in lung function in asthmatic patients with allergic bronchopulmonary mycosis

**DOI:** 10.1186/s13223-026-01024-2

**Published:** 2026-03-08

**Authors:** Yuka Kodama, Sachiko Takaoka, Takuya Nakashima, Kaho Matsunaga, Kosuke Terada, Yuga Yamashita, Hinako Masumitsu, Atsushi Miyasaka, Tatsuya Muraoka, Nami Masumoto, Takeshi Kaneko, Maiko Watanabe, Naomi Tsurikisawa

**Affiliations:** 1 Department of Respirology, National Hospital Organization Yokohama Medical Center, 3-60-2 Harajuku, Totsuka-ku, Yokohama, 245-8575 Japan; 2 Department of Pulmonology, Yokohama City University Graduate School of Medicine, 3-9 Fukuura, Kanazawa-ku, Yokohama, Kanagawa 236-0004 Japan; 3 Division of Microbiology, National Institute of Health Sciences, 3-25-26 Tonomachi, Kawasaki-ku, Kawasaki, Kanagawa 210-9501 Japan

**Keywords:** Allergic bronchopulmonary aspergillosis, Allergic bronchopulmonary mycosis, Aspergillus, IgE, Eosinophils

## Abstract

**Background:**

Allergic bronchopulmonary mycosis (ABPM), including allergic bronchopulmonary aspergillosis (ABPA), is an immune-mediated lung disease characterized by high serum total IgE and eosinophilia. The serum total IgE level is a well-established diagnostic and monitoring marker for ABPM/ABPA; however, its prognostic significance has not been fully elucidated. In particular, whether serum total IgE level at diagnosis is associated with subsequent longitudinal decline in lung function is unknown. Here we evaluated whether serum total IgE level at diagnosis is predictive of lung function decline and disease exacerbation in ABPA/ABPM.

**Methods:**

We retrospectively analyzed 39 patients diagnosed with ABPA/ABPM from April 2022 through February 2025. Clinical data, lung function, exacerbation rates (no./y), and treatment were assessed at diagnosis, 1 y afterward, and at final follow-up (mean, 4.7 y). Patients were classified according to IgE level at 1 y (dichotomized to ≥ 50% IgE reduction and < 50% IgE reduction).

**Results:**

During the study period, 100% of patients received inhaled corticosteroid, 56% received systemic corticosteroids, and 23% received antifungal therapy, but none was treated with any biologic. The exacerbation rate was correlated with serum IgE level at diagnosis (*p* = 0.026, *r* = 0.36) but not with eosinophilia. The serum IgE level at diagnosis was inversely correlated with change in percent vital capacity (*P* = 0.0015, *r *= –0.68), percent forced vital capacity (%FVC; *P* = 0.009, *r *= –0.70), and percent forced expiratory volume in 1 s (%FEV_1_; *P* = 0.038, *r *= – 0.48). Exacerbation rates at 1 y after diagnosis (*p* = 0.03) and final follow-up (*p* = 0.011) were lower in the IgE-decreased group than IgE-unchanged/increased group. The three patients with chronic pulmonary fibrosis were the only patients who showed consistently high serum total IgE levels and low pulmonary function (%FVC and %FEV₁) at diagnosis, as confirmed by computed tomography. In contrast, high-attenuation mucus, bronchiectasis, and mucus plugging showed no consistent relationship between serum total IgE levels and pulmonary function.

**Conclusion:**

High serum IgE levels at diagnosis may predict a decline in lung function and increased exacerbation risk in patients with ABPA/ABPM. Management of serum IgE levels may mitigate ABPA/ABPM exacerbation and help preserve pulmonary function.

## Background

Allergic bronchopulmonary mycoses are a hypersensitivity disorder of the airways and are characterized by type I (IgE-mediated) and type III (immune complex-mediated) allergic responses to colonization by filamentous fungi. The prototypical and most extensively studied form is allergic bronchopulmonary aspergillosis (ABPA), caused predominantly by *Aspergillus fumigatus *[1–5] but also including *Aspergillus* species such as *A. versicolor*, *A. niger*, *A. ochraceus*, *A. oryzae*, *A. terreus*, and *A. flavus *[1, 6].

In addition, allergic bronchopulmonary mycosis (ABPM) refers to disease caused by fungi other than *Aspergillus* species [1].

Among available biomarkers, serum total IgE historically has been central to both the diagnosis and longitudinal assessment of ABPM [7]. When concurrent with indicative clinical, radiologic, and immunologic features, high baseline IgE levels strongly support the diagnosis of ABPM [1, 8]. High serum IgE is associated with high rates of hospitalization due to exacerbation [1] or decreased lung function (e.g., forced expiratory volume in 1 s [FEV_1_]) and worsened respiratory quality of life [9]. Consequently, timely diagnosis of ABPA/ABPM and treatment that targets inflammation are important to prevent irreversible pulmonary damage [10]. In one cohort study, both total and *A. fumigatus*-specific IgE levels were useful not only for monitoring treatment response but also for detecting disease exacerbation [7]. In 149 ABPA patients treated with combined corticosteroids and antifungals, the total IgE concentration dropped significantly during the acutely after but then rose again at exacerbation, [11] highlighting the dual utility of monitoring IgE fluctuations for both tracking disease activity and guiding therapy.

Only a few studies have examined the long-term pulmonary function trajectory in patients with ABPA/ABPM [12]. In one early study, percent vital capacity (%VC) decreased over time in 23 patients with ABPA, suggesting that disease duration was associated with a decline in pulmonary function [13]. The percent predicted FEV₁ was lower in patients with sensitization to *A. fumigatus*, including those with ABPA, than in those with non-sensitized asthma [9]. Elevated total serum IgE levels were negatively correlated with the rate of change in %FEV₁ in 102 patients with cystic fibrosis and ABPA [14]. Similarly, annual decline in FEV₁ was more rapid in patients with non-ABPA/ABPM asthma who experienced at least two exacerbations annually compared with those experiencing less frequent exacerbations [15]. Even with systemic corticosteroid treatment, long-term complete remission of ABPA/ABPM is rarely achieved; instead, patients tend to follow a relapsing–remitting course, with intermittent exacerbation [12] and progression to chronic corticosteroid dependency [ 16].

Clinical management of ABPA combines the elimination of environmental sources of mold with pharmacological therapy. First-line treatment for disease exacerbation is moderate-dose oral corticosteroids [1, 5, 17]. A 4-month course of an azole antifungal agent, either itraconazole [18] or voriconazole [19], is recommended as second-line treatment [1]. Biologics targeting type 2 immune responses, IgE, or eosinophils have been used in patients who have severe asthma complicated by ABPA and in whom standard therapy has failed to achieve long-term remission [17, 20, 21].

The factors independently predictive of risk of exacerbation in ABPA include a peripheral blood eosinophil count > 1000/µL, degree of bronchiectasis, female sex, and younger age at onset. In addition, the on-treatment dynamics of serum total IgE levels are prognostic: a 20% decrease in the IgE level predicts a good response to therapy, whereas a 50% increase predicts exacerbation [22]. However, the extent to which total serum IgE levels at diagnosis and post-treatment changes in IgE levels affect long-term alterations in pulmonary function is unknown.

Here we assessed the outcomes of conventional treatment with systemic corticosteroids, inhaled corticosteroids, and antifungal agents in ABPA/ABPM. We specifically examined whether the serum total IgE level at diagnosis is predictive of subsequent changes in pulmonary function. In the present study we excluded patients who had received biologic agents because we anticipated that the concomitant use of corticosteroid and antifungal therapy would complicate the analysis of their effects.

## Methods

### Patients

We retrospectively collected data on the clinical courses of the 39 asthmatic patients who were diagnosed with ABPA/ABPM from April 2022 through February 2025 and treated at the Department of Respirology, National Hospital Organization Yokohama Medical Center (Kanagawa, Japan). Patients were diagnosed with ABPA/ABPM when they fulfilled at least 6 of 10 previously proposed criteria: [23] (1) current or previous history of asthma or asthmatic symptoms; (2) peripheral blood eosinophilia (≥ 500 cells/mm^3^); (3) elevated total serum IgE levels (≥ 417 IU/mL); (4) immediate cutaneous hypersensitivity or specific IgE for filamentous fungi; (5) the presence of precipitins or specific IgG for filamentous fungi; (6) filamentous fungal growth from sputum cultures or bronchial lavage fluid; (7) the presence of fungal hyphae in bronchial mucus plugs; (8) central bronchiectasis on computed tomography (CT); (9) the presence of mucus plugs in central bronchi, based on CT/bronchoscopy or mucus plug expectoration history; and (10) high-attenuation mucus (HAM) in the bronchi on CT.

Asthma was diagnosed and asthma severity was classified (stage 1, intermittent; stage 2, mild persistent; stage 3, moderate persistent; or stage 4, severe persistent) according to Global Initiative for Asthma guidelines [24].

The Ethics Committee of National Hospital Organization Yokohama Medical Center approved this retrospective review of existing medical records (Approval no. 2022-04), and written informed consent was obtained from all patients or their legal representatives.

## Study protocol

For this study, we assessed peripheral blood eosinophil counts, serum total IgE levels, pulmonary function tests, and exacerbation rates at four time points: 1 year before diagnosis, at ABPA/ABPM diagnosis, 1 y after diagnosis, and at the final follow-up visit. ABPA was defined as disease caused by *Aspergillus* species, whereas ABPM was due to any mold organism other than *Aspergillus* species [1]. Exacerbations were categorized according to three types: ABPA/ABPM exacerbations, asthma exacerbations, and infection-related exacerbations [1]. ABPA exacerbations were defined as the persistence of clinical symptoms for more than 2 weeks and the appearance of new infiltrates on chest imaging after the serum total IgE level increased by ≥ 50%. Asthma exacerbations were not associated with increased serum total IgE levels or new infiltrates but required a short course of systemic corticosteroids for management. Infective exacerbations were defined as those with bacterial growth from the sputum but without elevation in the serum total IgE level [1]. We separately analyzed each type of exacerbation as well as the total number of all three types combined. Exacerbation rates were calculated as the number of exacerbations divided by the observation period (in years) since the diagnosis of ABPA/ABPM.

## Measurement of eosinophils in peripheral blood, serum total IgE, and antigen-specific IgE

Patients’ peripheral blood eosinophils and WBC were counted by hemocytometry on their first hospital visit before the initiation of systemic corticosteroid treatment, and the ratio of eosinophils to WBC was calculated. The total serum IgE level (IU/mL) was measured by ELISA and by the nephelometry method using Elecsys IgE II (Cobas-e411; Roche Diagnostics, Tokyo, Japan) [25]. The intra- and inter-assay coefficients of variation were 2.5% and 6.0%, respectively. In accordance with the Japanese diagnostic criteria [23], we adopted 417 IU/mL as the disease-specific cut-off when ABPM-related analyses were performed.

In addition, the CAP-RAST method [26] was used to measure antigen-specific IgE levels (IU/mL) for nine different allergens: house dust mite (indicated as *Dermatophagoides pteronyssinus*, *Dermatophagoides farina*, or mix), cat, *Aspergillus*, *Alternaria*, *Penicillium*, *Cladosporium*, *Candida*, *Pityrosporum*, and *Trichophyton*.

We divided the study population into 2 groups according to the total serum IgE level at 1 y after diagnosis of ABPA/ABPM. The serum total IgE decreased group was defined as patients whose total serum IgE levels at 1 y after diagnosis of ABPA/ABPM were less than half the value at the time of ABPA/ABPM diagnosis. Patients comprising the serum total IgE unchanged/increased group were those whose total serum IgE levels at 1 y after diagnosis of ABPA/ABPM remained at least equal to half the value at diagnosis.

### Measurement of IgG against *Aspergillus* or precipitating antibodies against other molds

Serum IgG levels specific to *A. fumigatus* were measured using ELISA [27]. Ouchterlony double-immunodiffusion testing [28] of patient serum confirmed the presence of antigen-specific precipitating antibodies to multiple *Aspergillus* species (*A. fumigatus*, *A. flavus*, *A. glaucus*, *A. niger*, *A. restrictus*, and *A. versicolor* [Greer, Lenoir, North Carolina, USA], and *A. fumigatus* [Torii Pharmaceutical, Tokyo, Japan]). In select cases, precipitating antibodies to *Schizophyllum commune* were measured also using an in-house assay at the National Hospital Organization Sagamihara Hospital.

## Culture and identification of fungal species

Sputum and bronchial lavage samples were cultured in-house on potato dextrose agar at 25 °C for 7–14 days; resulting colonies were identified using morphologic evaluation [29] and molecular methods. Specifically, partial sequences of the β-tubulin gene obtained using the primers Bt2a and Bt2b [30] underwent BLAST analysis. Cultured isolates were identified at the National Institute of Health Sciences, Japan.

## Assessment of lung function

Predicted values for spirometric indices (FEV₁ and forced vital capacity [FVC]) were calculated using the Global Lung Function Initiative 2012 reference Eq. [31] Data are presented “% predicted”, calculated as follows: measured/predicted × 100%. VC, FVC, FEV₁, maximum expiratory flow at 50% of FVC (V₅₀) and at 25% of FVC (V₂₅) were measured using spirometry (DISCOM-51;, CHEST, Tokyo, Japan). Again, parameters are presented as a percentage of the predicted value (%VC, %FVC, %FEV₁, %V_50_, and %V_25_, respectively).

### Imaging findings

Seropositive ABPA/ABPM was defined as patients with positive antigen-specific IgG or precipitating antibodies but no abnormal findings on CT, including bronchiectasis, HAM, mucus plugging, and chronic pulmonary fibrosis (CPF) [1]. Bronchiectasis was defined as airway dilation exceeding the diameter of the accompanying pulmonary artery. HAM was defined as mucus showing higher attenuation than adjacent paraspinal muscle. Mucus plugs were identified as tubular or branching opacities within the airways. CPF was defined as evidence of fibrotic changes such as reticulation, traction bronchiectasis, and volume loss. The severity of bronchiectasis on CT was semiquantitative evaluated using a modified Reiff scoring system [32].

## Treatment

Treatment with or without systemic corticosteroid administration was recorded. The daily dose of inhaled corticosteroid was converted to an equivalent dose of fluticasone propionate. The use of long-acting β_2_-agonists, leukotriene receptor antagonists, long-acting muscarinic antagonists, sustained-release theophylline, and antifungal agents was also recorded. All patient data predated the initiation of biologic agents; therefore, none of the patients received biologic therapy during the study period.

### Statistical analysis

All values are expressed as the mean ± 1 SD, unless otherwise specified. Data were compared among groups using two-way analysis of variance according to a repeated-measures algorithm, followed by post hoc comparison using the Newman–Keuls test. The two mean values obtained by this process were compared using the Wilcoxon matched-pairs *t-*test. Correlation coefficients were obtained by using Spearman’s rank correlation test. Multiple logistic regression analysis was used to calculate risk factor coefficients. *P* < 0.05 was considered statistically significant. Statistical analyses were performed using SPSS for Windows, version 29 (SPSS, Chicago, Illinois, USA).

## Results

### Clinical findings and treatments

Among the 39 asthmatic patients with ABPA/ABPM who comprised our study population, 10 were male (Table 1). The mean age at asthma onset was 44.3 y, and the mean age at diagnosis of ABPA/ABPM (ABPA, 25 patients; ABPM, 14 patients) was 63.6 y. The causative organism in the 14 patients with ABPM included *A. versicolor* in five patients and *S. commune* in one patient, with positivity for two, three, or four *Aspergillus* species in one patient each; the causative organism could not be identified in five patients. ABPA/ABPM was diagnosed according to the Japanese diagnostic criteria [23], which do not require identification of the causative fungal organism when sufficient clinical, radiological, and immunological criteria are met. At diagnosis of ABPA/ABPM, the mean peripheral blood eosinophil count was 1426.5 ± 1087.5 cells/µL, and the median total serum IgE level was 916.0 IU/mL (range 65.3–13,167 IU/mL; Table 2).

**Table 1:** Characteristics of the 39 asthmatic patients with ABPA/ABPM

Age at entry of study (y)	68.3 ± 12.7
Sex (male/female)	10 / 29
Age at onset asthma (y)	44.3 ± 22.6
Onset type: childhood onset / adult recurrence / adult onset	6 / 1 / 32
Disease type: ABPA / ABPM	34 / 5
Age at diagnosis of ABPA / ABPM (y)	63.6 ± 14.6
Duration of asthma (from onset to study entry, y)	4.7 ± 5.8
Time from onset of asthma to diagnosis of ABPA/ABPM (y)	19.3 ± 15.5
*Lung function*	
Change in %VC (mL/y)	18.4 ± 54.0
Change in %FVC (mL/y)	14.6 ± 50.3
Change in %FEV_1_ (mL/y)	24.1 ± 66.1
*Exacerbations*	
Combined exacerbations: asthma, infection, and ABPA/ABPM	
During 1 y before diagnosis (no.)	3.1 ± 3.8
During 1 y after diagnosis (no.)	2.1 ± 2.2
During the 1 y before final follow-up (no.)	2.5 ± 3.1
Exacerbation rate	
Asthma (no./y)	1.15 ± 1.69
Infection (no./y)	0.04 ± 0.20
ABPA/ABPM (no./y)	0.532 ± 0.50
Overall exacerbation rate (no./y)	1.75 ± 1.70
*Treatment*	
Systemic corticosteroid, yes / no (%)	22 / 17 (56.4%)
Daily prednisolone dose (mg)	23.7 ± 10.6
Daily dose of inhaled corticosteroid (µg; converted to fluticasone equivalent)	789.2 ± 301.6
LABA, yes / no (%)	35 / 4 (89.7%)
LTRA, yes / no (%)	28 / 11 (71.8%)
LAMA, yes / no (%)	10 / 29 (25.6%)
SRN, yes / no (%)	12 / 27 (30.8%)
Antifungal drugs, yes / no (%)	9 / 30 (23.1%)
Biologics, yes / no (%)	0 / 39 (0%)
*CT findings*	
Seropositive / bronchiectasis / mucus plugging / HAM / CPF	0 / 5 / 1 / 30 / 3

Unless indicated otherwise, data are given as no. of patients or mean ± 1 SD. CPF, chronic pulmonary fibrosis

**Table 2:** Lung function and peripheral blood analyses in the 39 patients with ABPA/ABPM

Peripheral blood analysis	At diagnosis	At 1 y after diagnosis	At last examination	*P* value: diagnosis vs. 1 y	*P* value: diagnosis vs. last
	*N* = 39	*N* = 38	*N* = 23		
Log_10_ IgE RIST in serum (IU/mL)	3.078 ± 0.524	2.963 ± 0.576	3.006 ± 0.557	0.03	0.011
No. eosinophils (cells/mL)	1426.1 ± 1087.4	749.2 ± 697.2	784.0 ± 765.0	0.16	0.74
Lung function	*N* = 39	*N* = 36	*N* = 20		
%VC	96.5 ± 23.8	103.3 ± 23.2	97.9 ± 27.9	0.02	0.14
%FVC	92.1 ± 23.0	100.2 ± 22.1	91.7 ± 26.0	0.004	0.34
%FEV_1_	79.0 ± 24.5	88.1 ± 24.9	82.8 ± 28.0	0.003	0.10
FEV_1_/FVC (%)	68.1 ± 12.0	69.8 ± 10.9	70.6 ± 11.8	0.12	0.15
%V50	37.3 ± 23.9	43.4 ± 22.3	40.6 ± 21.3	0.04	0.34
%V25	38.0 ± 23.4	35.3 ± 23.4	37.6 ± 21.3	0.34	0.10

Unless indicated otherwise, data are given as the mean ± 1 SD. Note, lung function parameters are presented as a percentage of the predicted value

At the diagnosis of ABPA/ABPM, pulmonary function tests showed preserved %VC and %FVC (both with mean values > 80%), whereas the mean %FEV₁ was 79.0% and the mean FEV₁/FVC ratio was 68.1%, indicating obstructive ventilatory impairment. At the final follow-up visit, mean %FEV₁ had improved to 82.8% overall (Table 2). The overall exacerbation rate was 3.1 episodes per year during the year prior to diagnosis of ABPA/ABPM; this rate decreased to approximately 2.5 episodes annually for the final year of follow-up. Asthma exacerbations were the most frequent (1.15 annually), followed by ABPA/ABPM exacerbations (1.04 annually), whereas infection-related exacerbations were rare (0.532 annually; Table 1).

Systemic corticosteroids were administered to 56.4% of patients, with a mean daily prednisolone dose of 23.7 mg. The average daily dose of inhaled corticosteroids, converted to the equivalent dose of fluticasone propionate, was 789.2 µg/day. Antifungal agents were used in 23.1% of patients. Chest CT revealed bronchiectasis in five patients, mucus plugging in one, HAM in 30, and CPF in three. No patients in the present study had seropositive ABPA (Table 1).

There were no significant differences between the IgE-decreased and IgE-unchanged/increased groups in the proportion of patients with ABPM, age, smoking history, comorbidities, total serum IgE level, or peripheral blood eosinophil count at ABPA/ABPM diagnosis (Tables 3 and 4). However, peripheral blood eosinophil counts at the final follow-up visit were lower in the IgE-decreased group (*P* = 0.014; Table 3). Pulmonary function values at diagnosis of ABPA/ABPM and at the final follow-up did not differ between groups (Table 4).

**Table 3:** Characteristics and therapy of patients of ABPA/ABPM according to serum IgE level

	Serum total IgE decreased group	Serum total IgE unchanged/ increased group	
	*N* = 11	*N* = 27	*P* value
Age at study entry (y)	68.2 ± 16.1	68.9 ± 11.4	0.87
Sex (male/female)	1 / 10	8 / 19	0.18
Age at onset of asthma (y)	38.5 ± 22.1	46.7 ± 23.1	0.32
Onset type: childhood onset / adult recurrence / adult onset	3 / 0 / 8	3 / 1 / 23	0.40
Type: ABPA / ABPM	10 / 1 (90.9%)	23 / 4 (85.2%)	0.96
Age at diagnosis of ABPA / ABPM (y)	61.6 ± 17.5	64.7 ± 13.6	0.56
Duration of asthma (from onset to study entry; y)	23.2 ± 13.7	18.1 ± 16.4	0.37
Time from onset of asthma to diagnosis of ABPA/ABPM (y)	6.5 ± 6.5	4.2 ± 5.4	0.26
Smoking history, never / ex /current	10 / 1 / 0	22 / 5 / 0	0.65
Comorbidities			
Allergic rhinoconjunctivitis, yes / no	6 / 4	14 / 11	0.87
Atopic dermatitis, yes / no	3 / 7	4 / 20	0.39
*Peripheral blood analysis*			
At diagnosis, *N *= 39			
log IgE RIST in serum (IU/mL)	3.185 ± 0.608	3.050 ± 0.496	0.48
No. eosinophils (cells/mL)	1600.8 ± 1232.4	1397.8 ± 1047.0	0.60
*At 1 y after diagnosis, N* = 38			
log IgE RIST in serum (IU/mL)	2.690 ± 0.651	3.078 ± 0.511	0.06
Number of eosinophils (cells/mL)	507.9 ± 566.7	847.5 ± 730.4	0.18
*At final follow-up, N* = 23			
log IgE RIST in serum (IU/mL)	2.728 ± 0.700	3.084 ± 0.507	0.21
Number of eosinophils (cells/mL)	212.2 ± 189.5	942.8 ± 791.9	0.014
*Lung function*			
*At diagnosis, N *= 39			
VC (predicted %) (%)	94.6 ± 25.1	97.4 ± 24.2	0.76
FVC (predicted %) (%)	92.5 ± 25.9	92.0 ± 22.7	0.95
FEV_1_ (predicted %) (%)	77.5 ± 23.6	79.5 ± 25.7	0.82
FEV_1_/FVC (%)	65.9 ± 16.2	69.0 ± 10.0	0.47
V50 (predicted %) (%)	38.5 ± 30.4	36.2 ± 21.5	0.80
V25 (predicted %) (%)	30.0 ± 15.8	41.1 ± 39.8	0.38
*At 1 year after diagnosis, N* = 36			
%VC	107.6 ± 15.5	101.7 ± 25.6	0.49
%FVC	103.4 ± 16.2	98.9 ± 24.1	0.59
%FEV_1_	90.5 ± 23.6	87.2 ± 25.7	0.73
FEV_1_/FVC (%)	71.3 ± 16.3	69.3 ± 8.7	0.64
%V50	52.9 ± 30.6	39.7 ± 17.6	0.11
%V25	36.3 ± 19.9	34.9 ± 25.0	0.88
*At final follow-up, N *= 20			
%VC	97.1 ± 25.1	98.2 ± 29.6	0.94
%FVC	89.9 ± 25.6	92.3 ± 27.1	0.87
%FEV_1_	75.3 ± 26.9	85.3 ± 28.9	0.51
FEV_1_/FVC (%)	65.6 ± 21.3	71.9 ± 8.5	0.35
%V50	40.4 ± 25.7	40.7 ± 20.7	0.98
%V25	31.4 ± 13.4	39.6 ± 23.4	0.47
*Changes in lung function*			
Change in %VC (mL/y)	38.8 ± 54.5	10.3 ± 52.6	0.16
Change in %FVC (mL/y)	21.2 ± 37.0	12.0 ± 55.0	0.63
Change in %FEV_1_ (mL/y)	47.4 ± 98.0	15.1 ± 48.5	0.19
*Combined exacerbations: asthma, infection, and ABPA/ABPM*			
During 1 y before diagnosis (no.)	3.18 ± 3.13	3.07 ± 4.13	0.94
During 1 y after diagnosis (no.)	1.36 ± 1.21	2.44 ± 2.41	0.17
During 1 y before final follow-up (no.)	0.91 ± 0.94	3.19 ± 3.48	0.004
Exacerbation rates			
Asthma (no./y)	1.04 ± 1.37	1.23 ± 1.84	0.76
Infection (no./y)	0.11 ± 0.30	0.05 ± 0.14	0.41
ABPA/ABPM (no./y)	0.50 ± 0.44	0.56 ± 0.54	0.72
Overall exacerbation rate (no./y)	1.65 ± 1.69	1.85 ± 1.75	0.75
*Treatment*			
Systemic corticosteroid, yes / no (%)	7 / 4 (63.6%)	15 / 12 (55.6%)	0.65
Daily prednisolone dose (mg)	20.7 ± 6.1	25.1 ± 12.1	0.38
Daily dose of ICS (mg; converted to fluticasone equivalent)	710.0 ± 317.8	811.5 ± 299.8	0.38
LABA, yes / no (%)	9 / 2 (81.8%)	25 / 2 (92.3%)	0.33
LTRA, yes / no (%)	7 / 4 (63.6%)	20 / 7 (74.0%)	0.52
LAMA, yes / no (%)	2 / 9 (18.2%)	8 / 19 (29.6%)	0.47
SRN, yes / no (%)	2 / 9 (18.2%)	9 / 18 (33.3%)	0.35
Antifungal drugs, yes / no (%)	4 / 7 (36.4%)	5 / 22 (18.5%)	0.24
Biologics, yes / no (%)	0 / 11 (0%)	0 / 28 (0%)	–
*CT findings*			
Modified Reiff score	9.0 ± 3.8	8.9 ± 3.6	0.91
Seropositive / bronchiectasis / mucus plugging / HAM / CPF	0 / 1 / 1 / 8 / 1	0 / 4 / 0 / 21 / 2	0.44

Unless indicated otherwise, data are given as the no. of patients or mean ± 1 SD. Note, lung function parameters are presented as a percentage of the predicted value. CPF, chronic pulmonary fibrosis; ICS, inhaled corticosteroid

**Table 4:** Lung function and peripheral blood analysis in patients with ABPA/ABPM according to change in serum IgE level

Peripheral blood analysis	At diagnosis	At 1 y after diagnosis	At last examination	*P* value: At diagnosis vs. at 1 y after diagnosis	*P* value: At diagnosis vs. at final follow-up
	*N* = 39	*N* = 38	*N* = 23		
*Serum total IgE decreased group*					
Log IgE RIST in serum (IU/mL)	3.185 ± 0.608	2.690 ± 0.651	2.728 ± 0.700	0.001	0.018
No. eosinophils in WBC (cells/mL)	1600.8 ± 1232.4	507.9 ± 566.7	212.2 ± 189.5	0.008	0.09
*Serum total IgE unchanged/increased group*					
Log IgE RIST in serum (IU/mL)	3.050 ± 0.496	3.078 ± 0.511	3.084 ± 0.507	0.64	0.94
No. eosinophils (cells/mL)	1397.8 ± 1047.0	847.5 ± 730.4	942.8 ± 791.9	0.002	0.009
Lung function	*N* = 39	*N* = 36	*N* = 20		
*Serum total IgE decreased group*					
%VC	94.6 ± 25.1	107.6 ± 15.5	97.1 ± 25.1	0.047	0.99
%FVC	92.5 ± 25.9	103.4 ± 16.2	89.9 ± 25.6	0.027	0.95
%FEV_1_	77.5 ± 23.6	90.5 ± 23.6	75.3 ± 26.9	0.044	0.71
FEV_1_/FVC (%)	65.9 ± 16.2	71.3 ± 16.3	65.6 ± 21.3	0.13	0.49
%V50	38.5 ± 30.4	52.9 ± 30.6	40.4 ± 25.7	0.09	0.73
%V25	30.0 ± 15.8	36.3 ± 19.9	31.4 ± 13.4	0.21	0.51
*Serum total IgE unchanged/increased group*					
%VC	101.7 ± 25.6	97.4 ± 24.2	98.2 ± 29.6	0.15	0.09
%FVC	98.9 ± 24.1	92.0 ± 22.7	92.3 ± 27.1	0.024	0.26
%FEV_1_ (predicted %) (%)	87.2 ± 25.7	79.5 ± 25.7	85.3 ± 28.9	0.029	0.09
FEV_1_/FVC (%)	69.3 ± 8.7	69.0 ± 10.0	71.9 ± 8.5	0.54	0.49
%V50	39.7 ± 17.6	36.2 ± 21.5	40.7 ± 20.7	0.23	0.38
%V25	34.9 ± 25.0	41.1 ± 39.8	39.6 ± 23.4	0.73	0.15

Unless indicated otherwise, data are given as no. of patients or mean ± 1 SD. Note, lung function parameters are presented as a percentage of the predicted value

Although overall exacerbation rates for the year before diagnosis and the first year after diagnosis were similar between groups, the overall exacerbation rate for the final year of observation was lower (*P* = 0.004) among patients in the IgE-decreased group (Table 3). Analysis of exacerbations by category, namely asthma, ABPA/ABPM, and infection, revealed no significant differences between the two groups. There were no significant differences between the IgE-decreased and IgE-unchanged/increased groups regarding systemic corticosteroid dose, antifungal therapy, or other concomitant asthma medications. Likewise, neither CT findings nor the severity of bronchiectasis according to a modified Reiff score [32] differed between the patient groups (Table 3).

In logistic regression analysis examining factors associated with a reduction in serum total IgE to less than half at 1 y after treatment initiation, %FVC at diagnosis showed a weak but statistically significant association (odds ratio 0.941; *P* = 0.038). In contrast, no significant associations were observed for treatment modalities, including oral corticosteroids, inhaled corticosteroids, and antifungal agents (Table 5).

**Table 5:** Logistic regression analysis of baseline factors predictive of a decrease in serum total IgE

	Odds ratio	95% confidence interval	*P* value
%FVC at diagnosis	0.941	0.888–0.996	0.038
%FEV_1_ at diagnosis	1.026	0.992–1.062	0.14
Systemic corticosteroids	1.212	0.250–5.875	0.61
Inhaled corticosteroids	0.249	0.010–6.033	0.39
Antifungal drugs	4.288	0.608–30.25	0.14

**Fig. 1:**
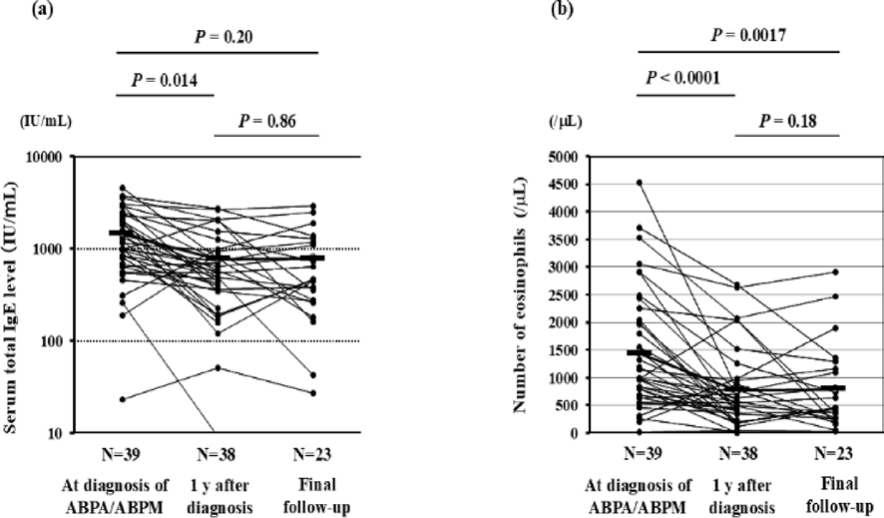
Longitudinal changes in total serum IgE levels and peripheral blood eosinophil count in asthmatic patients with ABPA/ABPM. **a** Total serum IgE level and **b** peripheral blood eosinophil count at diagnosis of ABPA/ABPM, at 1 year after diagnosis, and at final follow-up

**Fig. 2:**
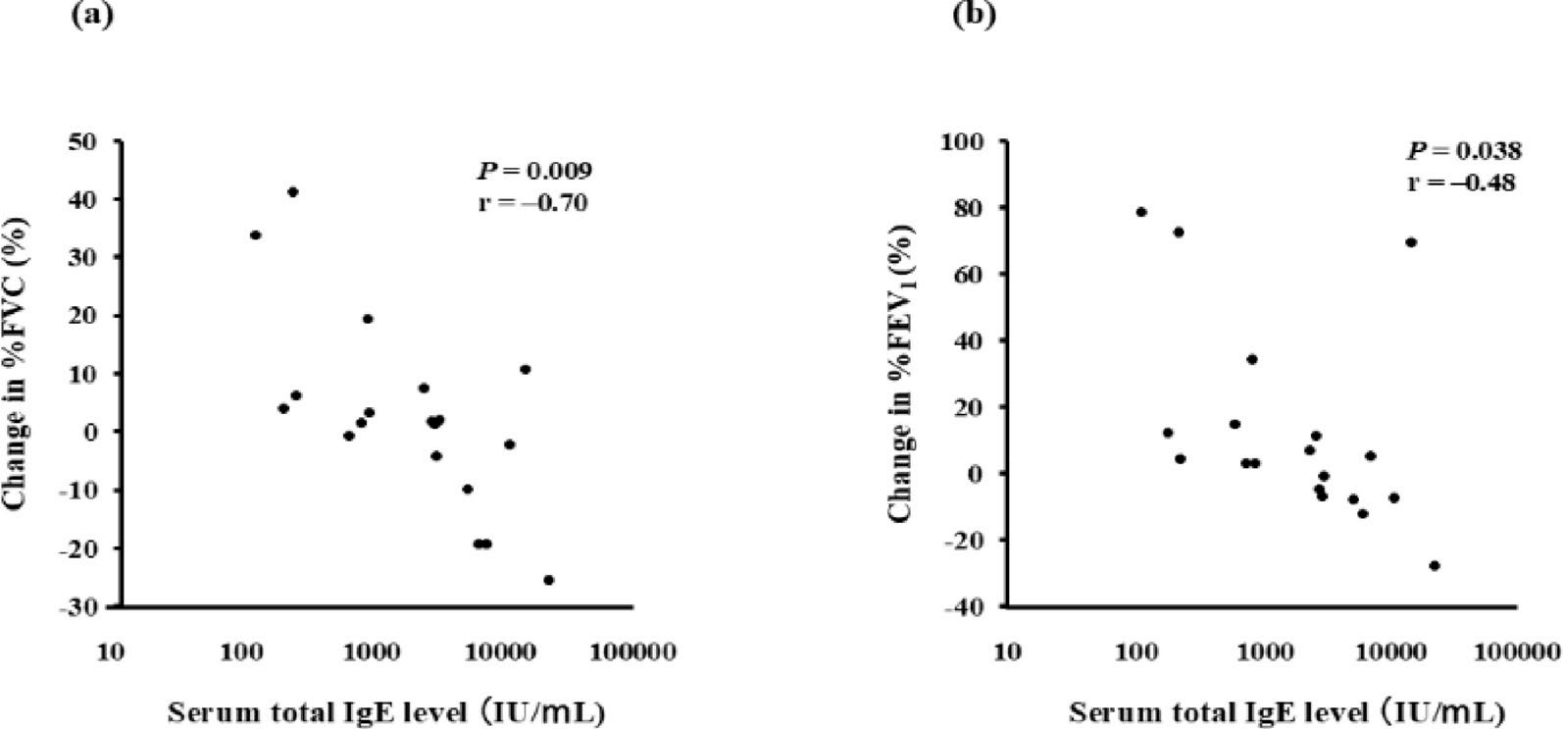
Correlation between total serum IgE levels at diagnosis of ABPA/ABPM and changes in pulmonary function. Correlation between total serum IgE level at diagnosis of ABPA/ABPM and the rate of change in **a** %FVC, and **b** %FEV₁. Pulmonary function changes were calculated using values obtained at diagnosis and at the final pulmonary function test. The rates of change in %VC, %FVC, and %FEV₁ were calculated using the following formula: (Value at final examination − Value at diagnosis of ABPA/ABPM) / Value at diagnosis of ABPA/ABPM × 100%. Longitudinal changes in pulmonary function were calculated using the following formula: (Value at final follow-up examination – Value at diagnosis of ABPA/ABPM) / time (y) since diagnosis of ABPA/ABPM

Compared with total serum IgE levels at the time of diagnosis of ABPA/ABPM, levels 1 y after diagnosis were significantly decreased (*P* = 0.014), whereas those at the final follow-up were not significantly different (Fig. 1a). However, peripheral blood eosinophil counts were lower at 1 y after diagnosis (*P* < 0.0001) and at the final follow-up (*p* = 0.0017) than at the time of diagnosis (Fig. 1b). Regarding pulmonary function, serum total IgE levels at diagnosis were negatively correlated with subsequent changes in %FVC (*P* = 0.009, *r* = − 0.70; Fig. 2a), and %FEV₁ (*P* = 0.038, *r* = − 0.48; Fig. 2b).

**Fig. 3:**
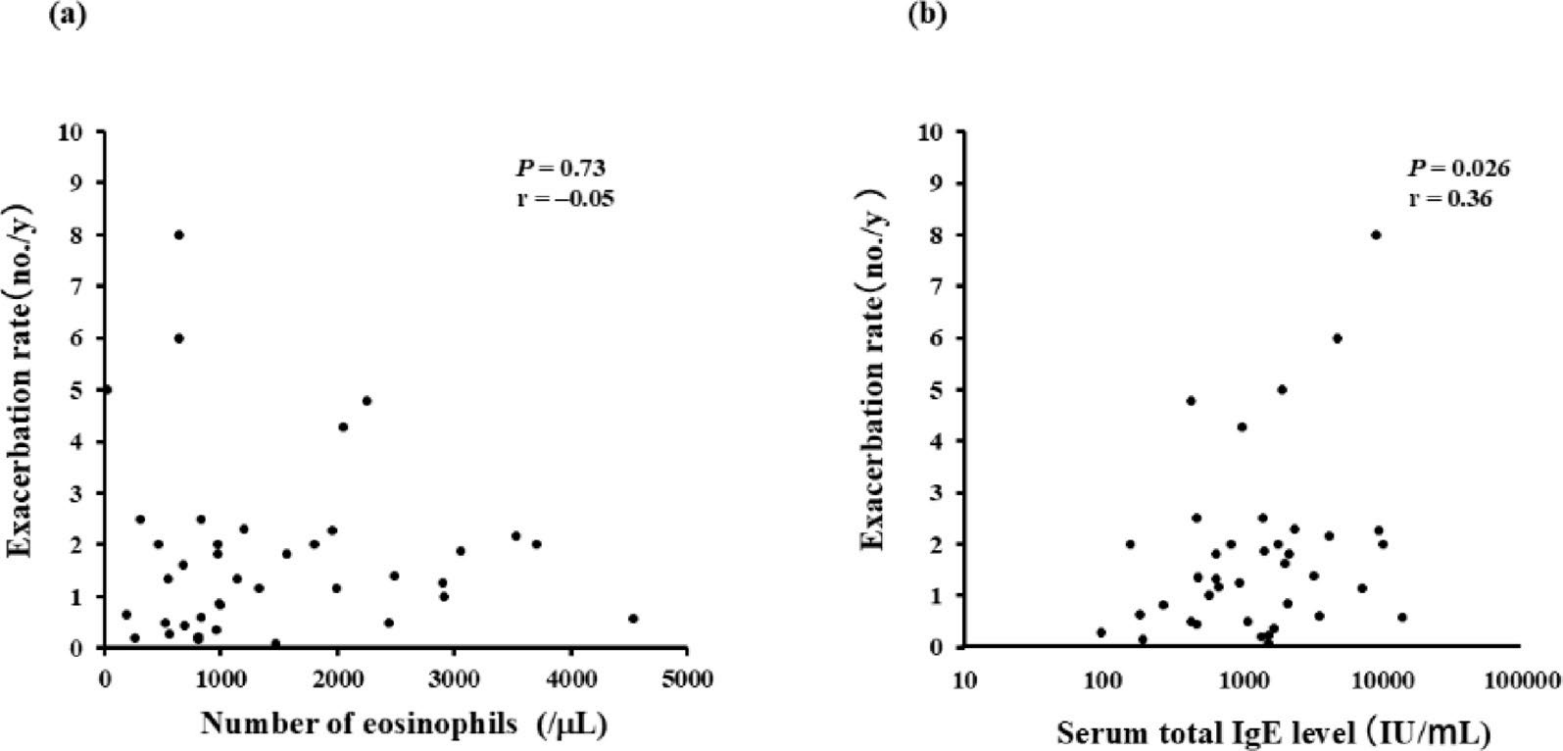
Correlation between the overall exacerbation rate and peripheral blood eosinophil count or total serum IgE level. Correlation between the overall exacerbation rate and **a** peripheral blood eosinophil count and **b** total serum IgE level at diagnosis. The exacerbation rate was calculated as the total number of exacerbations due to asthma, ABPA/ABPM, or infection divided by the observation period (y) from diagnosis of ABPA/ABPM to the final visit

**Fig. 4:**
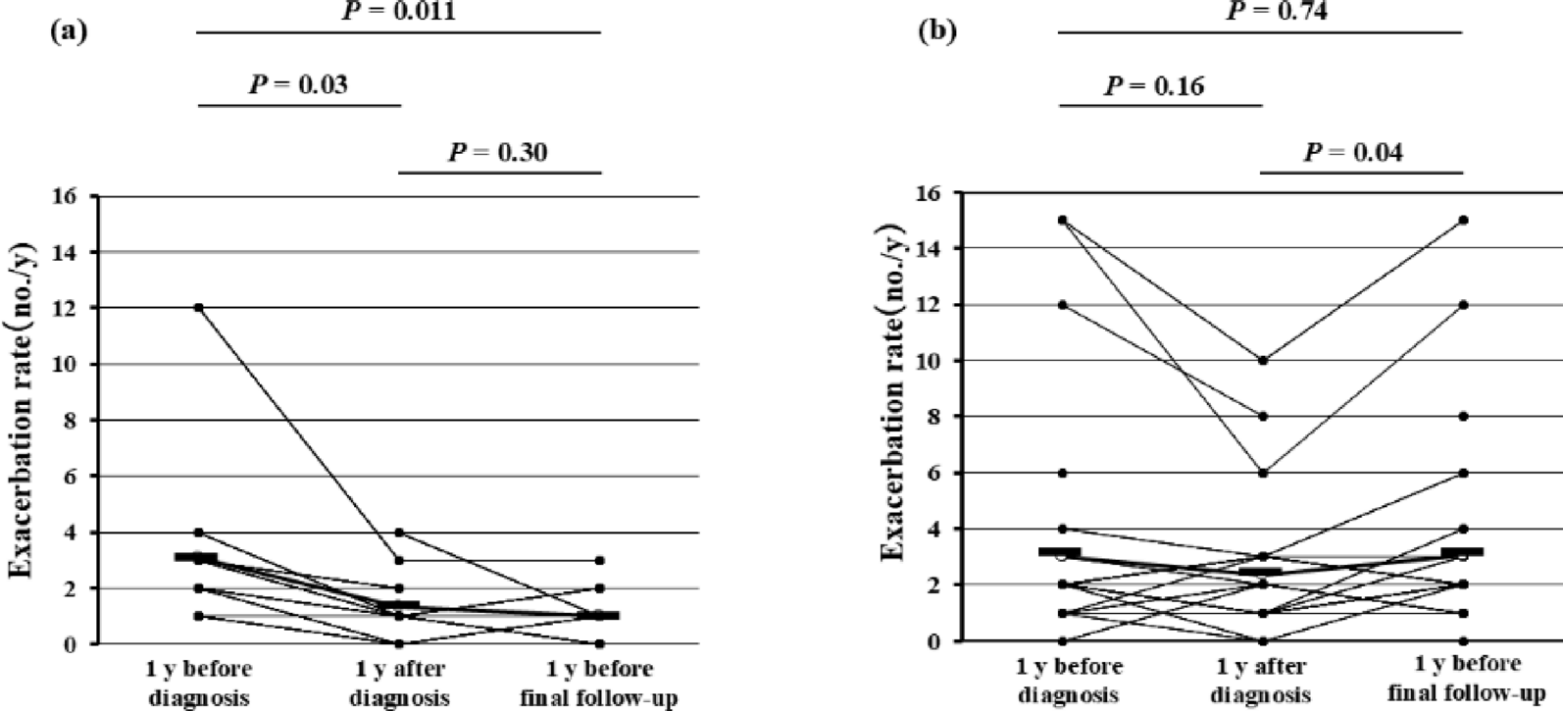
Overall exacerbation rate according to the reduction in total serum IgE level at 1 y after diagnosis of ABPA/ABPM. Patients were allocated into two groups: **a** those whose total serum IgE at 1 year after diagnosis of ABPA/ABPM was ≤ 50% of that at diagnosis (*n* = 11; decreased group) and **b **those whose was not (*n* = 28; unchanged/increased group). We then calculated the overall exacerbation rate for 3 intervals periods: 1 y before diagnosis of ABPA/ABPM, 1 y after diagnosis, and during the year before the last follow-up visit. We compared the resulting mean values by using the Wilcoxon matched-pairs *t-*test; *P* < 0.05 was considered statistically significant

The total exacerbation rate (no. of episodes/y) from diagnosis of ABPA/ABPM to the final follow-up visit included all disease flares due to asthma, ABPA/ABPM, and infection. This rate was not significantly correlated with the peripheral blood eosinophil count at diagnosis of ABPA/ABPM (Fig. 3a), but did show a positive correlation with the serum total IgE level at diagnosis (*P* = 0.026, *r* = 0.36; Fig. 3b). In the IgE-decreased group, the total exacerbation rate was lower at 1 y after diagnosis of ABPA/ABPM (*P* = 0.03) and during the final year of follow-up (*P* = 0.011) compared with the rate at the time of diagnosis (Fig. 4a). In contrast, total exacerbation rates did not vary over time in patients whose serum IgE level at 1 y after diagnosis was unchanged/increased (Fig. 4b).

**Fig. 5:**
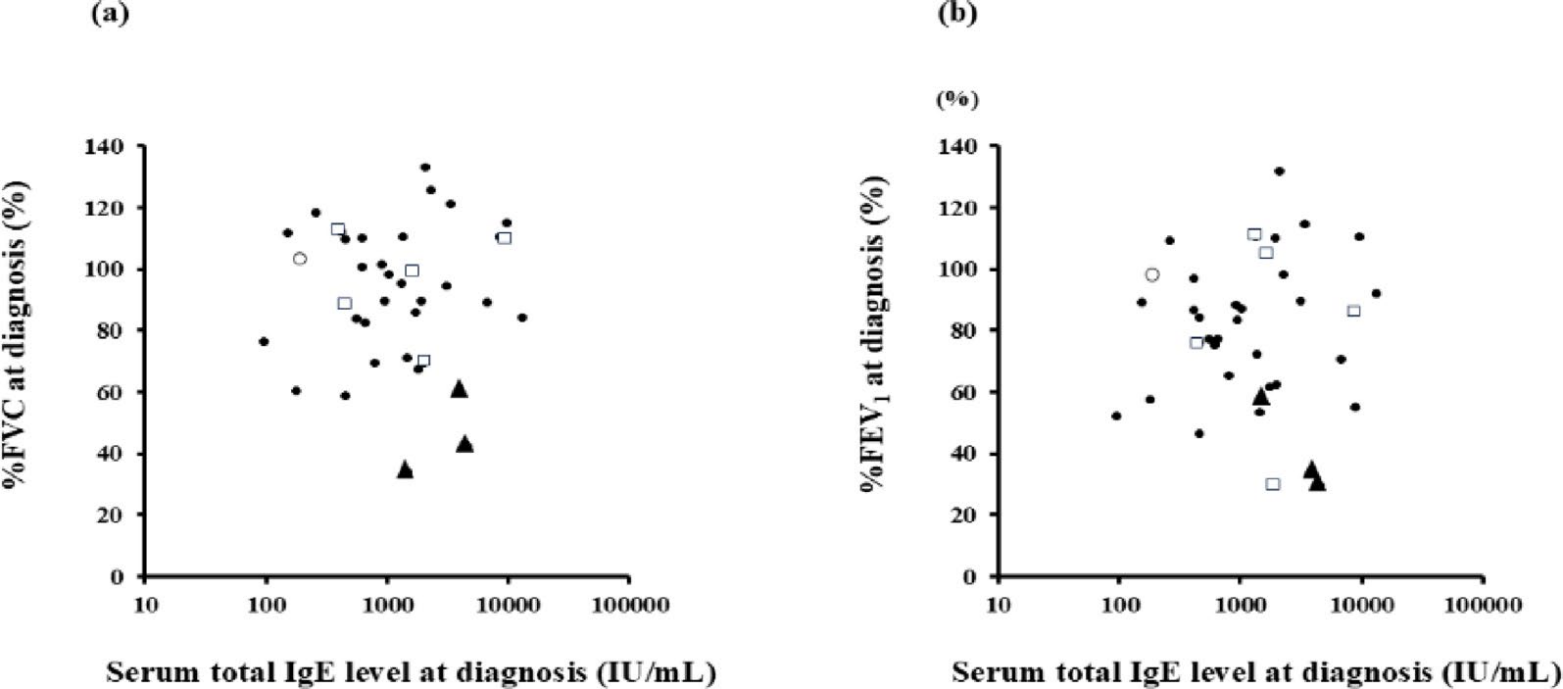
Correlation between %FVC at diagnosis of ABPA/ABPM, total serum IgE level, peripheral blood eosinophil count, and CT imaging features. Our analysis revealed a lack of significant correlation between %FVC at diagnosis of ABPA/ABPM and **a** total serum IgE level at diagnosis (*P* = 0.97, r = 0.01) or **b** peripheral blood eosinophil count at diagnosis (*P* = 0.95, *r* = 0.01). Patients showing CT findings characteristic of ABPA/ABPM are represented as follows: ○, mucus plugs; ●, high-attenuation mucus; ▲, chronic pulmonary fibrosis; □, bronchiectasis

At diagnosis of ABPA/ABPM, the total serum IgE level was not significantly correlated with %FVC (Fig. 5a) or %FEV₁ (Fig. 5b). When we included CT findings in the comparison, the three patients with CPF were the only ones who showed consistently high serum total IgE levels and low pulmonary function (%FVC and %FEV₁) at diagnosis. In contrast, there was no consistent relationship between serum total IgE levels and pulmonary function for patients with HAM, bronchiectasis, and mucus plugging.

## Discussion

In the present study, we investigated asthmatic patients who developed ABPA or ABPM prior to the introduction of biologic therapy. Our findings demonstrate that, under conventional treatment, factors that influenced disease exacerbation were associated not with peripheral blood eosinophil counts at diagnosis but rather with total serum IgE concentration at diagnosis. Increased serum total IgE levels at the time of diagnosis were significantly correlated with subsequent declines in pulmonary function parameters, including %VC, %FVC, and %FEV₁. Furthermore, patients whose total serum IgE level at 1 y after initiation of therapy was lower than 50% of that at diagnosis of ABPA/ABPM showed sustained suppression of exacerbations compared with patients whose IgE levels remained unchanged or increased over time. These results suggest that achieving a reduction in total serum IgE may be an important management goal in asthmatic patients with ABPA/ABPM. Our findings imply that the total serum IgE level could play a crucial role in guiding the future selection of biologic agents for these patients. Although treatment was not standardized, the lack of difference in treatment intensity between groups supports the notion that changes in pulmonary function were not driven primarily by therapeutic differences.

We classified patients according to four CT features of ABPA/ABPM (bronchiectasis, mucus plugging, HAM, and CPF) and created scatter plots to show the relationship between serum total IgE level and %FVC or %FEV₁ at diagnosis for each of these features (Fig. 5). Although statistical analysis was not feasible because of the small sample size, all three patients with CPF (solid triangles in Fig. 5) appeared to share several clinical features, including a long interval from asthma onset to ABPA diagnosis, high serum total IgE levels at diagnosis, and relatively low %FVC and %FEV₁ values. These findings suggest that patients who develop ABPA/ABPM long after their asthma diagnosis and who show elevated serum total IgE levels may already have advanced airway remodeling at the time of diagnosis of ABPA/ABPM [11]. Our results support the findings of a previous study [13] that revealed a negative correlation between disease duration and %VC, as well as with those of another study [14] showing that elevated serum total IgE is associated with decreased %FEV₁. Furthermore, loss of airway reversibility was more prevalent among our HAM-positive patients than in previous HAM-negative patients, [33] suggesting that HAM positivity may indicate extant airway remodeling. In that context, the reductions in %VC and %FVC in our patients with high total serum IgE levels may reflect fungal colonization or associated structural changes.

The anti-IgE antibody omalizumab is the first biologic agent for the treatment of severe asthma and has been explored as a possible ABPA treatment through case reports, a meta-analysis [34], and a single small-scale randomized control trial [35]. In addition, mepolizumab, a neutralizing antibody against the eosinophil growth factor interleukin (IL) 5, and benralizumab, an antibody against the IL5 receptor (IL5R) alpha chain, have been the focus of several case reports [36–39]. Likewise, the potential ABPA/ABPM treatment efficacy of the anti-IL4 receptor alpha-chain antibody dupilumab and the anti-thymic stromal lymphoprotein antibody tezepelumab, both of which suppress type 2 inflammation in general, have been addressed only through a few case reports to date [20, 36, 40]. A meta-analysis of 86 studies involving 346 cases of ABPA demonstrated that omalizumab, dupilumab, and mepolizumab were associated with significant clinical benefits, including a reduction in exacerbations, decreased oral corticosteroid use, and lowered serum IgE levels [20]. Although the effects of biologic agents such as omalizumab and anti-IL5/IL5R antibodies are better understood now than previously, knowledge remains sparse overall. Directly comparing the five biologics available in Japan will be challenging but represents an important area for future ABPA/ABPM research.

In one retrospective multivariate cohort analysis, [41] prognostic indicators of ABPA/ABPM included older age, time to diagnosis, the presence of mucus plugs on CT, and decreased lung function. Patients with delayed diagnosis of ABPA/ABPM frequently have moderate to severe bronchiectasis, poorly controlled asthma and cystic fibrosis, recurrent exacerbation, progressive pulmonary dysfunction, and significantly decreased quality of life [42]. A retrospective analysis of vital outcomes in 61 patients with ABPA reported a mean survival of 114 months, a 1-y survival rate of 91.3%, and a 5-y survival rate of 83.8% [43]. However, few studies have followed ABPA/ABPM patients for more than 10 y, and analyses of long-term prognosis are unavailable.

Our study has several limitations. This was a single-center retrospective study with a relatively small cohort, which limits generalizability and precludes causal inference; therefore, the results should be interpreted with caution. In addition, heterogeneity in diagnostic criteria and treatment strategies for ABPA/ABPM across institutions may have influenced the findings. Moreover, the present study included only patients who had not received biologic therapy, and thus may not fully reflect current treatment practices. Future prospective, multicenter studies with larger cohorts are needed to validate our findings, including the prognostic value of pulmonary function in patients receiving biologic therapies for ABPA/ABPM.

## Conclusion

Together, the results of the present and previous studies suggest that serum total IgE at diagnosis is an important biomarker of disease activity and prognosis in patients with ABPA/ABPM. Its usefulness should be validated in multicenter large-scale prospective studies that include patients treated with biologic agents. Understanding this association could help identify high-risk patients early, guide individualized treatment, and improve clinical outcomes. In addition, clarifying the prognostic role of IgE in the Japanese ABPA/ABPM population without cystic fibrosis may provide insights relevant to disease management in similar patient populations worldwide, given that ABPA/ABPM disease patterns in patients without cystic fibrosis differ from those in cystic fibrosis–dominated cohorts.

## Data Availability

No datasets were generated or analysed during the current study.

## Abbreviations

ABPA: Allergic bronchopulmonary aspergillosis
ABPM: Allergic bronchopulmonary mycosis
CPF: Chronic pulmonary fibrosis
CT: Computed tomography
FEV_1_: Forced expiratory volume in 1 s
FVC: Forced vital capacity
HAM: High-attenuation mucus
V_25_: Maximal expiratory flow at 25% of FVC
V_50_: Maximal expiratory flow at 50% of FVC
VC: Vital capacity
